# Prevalence and Antimicrobial Susceptibility Patterns of Bacteria from Milkmen and Cows with Clinical Mastitis in and around Kampala, Uganda

**DOI:** 10.1371/journal.pone.0063413

**Published:** 2013-05-07

**Authors:** David Patrick Kateete, Usuf Kabugo, Hannington Baluku, Luke Nyakarahuka, Samuel Kyobe, Moses Okee, Christine Florence Najjuka, Moses Lutaakome Joloba

**Affiliations:** 1 Department of Medical Microbiology, School of Biomedical Sciences, Makerere University College of Health Sciences, Kampala, Uganda; 2 College of Veterinary Medicine and Biosecurity, Makerere University, Kampala, Uganda; University College Dublin, Ireland

## Abstract

**Background:**

Identification of pathogens associated with bovine mastitis is helpful in treatment and management decisions. However, such data from sub-Saharan Africa is scarce. Here we describe the distribution and antimicrobial susceptibility patterns of bacteria from cows with clinical mastitis in Kampala, Uganda. Due to high concern of zoonotic infections, isolates from milkmen are also described.

**Methodology/Principal Findings:**

Ninety seven milk samples from cows with clinical mastitis and 31 nasal swabs from milkmen were collected (one sample per cow/human). Fifty eight (60%) Gram-positive isolates namely Staphylococci (21), Enterococci (16), Streptococci (13), Lactococci (5), Micrococci (2) and Arcanobacteria (1) were detected in cows; only one grew *Staphylococcus aureus*. Furthermore, 24 (25%) coliforms namely *Escherichia coli* (12), *Klebsiella oxytoca* (5), *Proteus vulgaris* (2), *Serratia* (2), *Citrobacter* (1), *Cedecea* (1) and *Leclercia* (1) were identified. From humans, 24 Gram-positive bacteria grew, of which 11 were Staphylococci (35%) including four *Staphylococcus aureus*. Upon susceptibility testing, methicillin-resistant coagulase-negative staphylococci (CoNS) were prevalent; 57%, 12/21 in cows and 64%, 7/11 in humans. However, methicillin-resistant *Staphylococcus aureus* was not detected. Furthermore, methicillin and vancomycin resistant CoNS were detected in cows (*Staphylococcus hominis*, *Staphylococcus lugdunensis*) and humans (*Staphylococcus scuiri*). Also, vancomycin and daptomycin resistant Enterococci (*Enterococcus faecalis* and *Enterococcus faecium,* respectively) were detected in cows. Coliforms were less resistant with three pan-susceptible isolates. However, multidrug resistant *Klebsiella*, *Proteus*, *Serratia, Cedecea*, and *Citrobacter* were detected. Lastly, similar species grew from human and bovine samples but on genotyping, the isolates were found to be different. Interestingly, human and bovine *Staphylococcus aureus* were genetically similar (spa-CC435, spa-type t645 corresponding to ST121) but with different susceptibility patterns.

**Conclusions/Significance:**

CoNS, Enterococci, Streptococci, and *Escherichia coli* are the predominant pathogens associated with clinical bovine-mastitis in Kampala, Uganda. Multidrug resistant bacteria are also prevalent. While similar species occurred in humans and cows, transmission was not detected.

## Introduction

Bovine mastitis is the inflammation of the mammary gland often due to microorganisms that invade the udder, multiply and produce toxins that are harmful to the mammary tissue [1]. Mastitis is a global problem responsible for huge financial losses to dairy industries and economies at large due to poor milk quality, reduced milk yield and increased expenditure on treatment and sometimes death due to the disease itself or through culling of affected cows [1]. In Uganda, the situation is no better in that farmers incur heavy costs due to chemotherapy and reduced milk production [2], [3].

Bovine mastitis manifests either as subclinical, in which there’s no visible symptom, or clinical, in which visible symptoms do occur, varying from mild (flakes in milk, slight swelling of infected quarter) to severe (abnormal milk secretions, hot swollen quarter/udder, fever, rapid pulse, loss of appetite, depression and death) [1].

Subclinical mastitis is relatively well documented in Uganda and reports indicate that poor management as well as antimicrobial resistance aggravate the condition [2], [3]. While these important studies demonstrate a growing problem of mastitis, there’s scanty data on clinical mastitis in this country. Although subclinical mastitis is economically more important to the dairy industry, most farmers in Uganda are ignorant of it (due to concealed symptoms) [2], [3] but are aware of clinical mastitis, probably due to the apparent symptoms which they perceive as an imminent threat to cows. Besides, clinical mastitis is also of considerable importance in that it causes both animal suffering and economic loss [4].

The effective control of mastitis heavily relies on husbandry and management practices [1]; however, the identification of associated pathogens may be helpful in treatment and in making sound management decisions [5], [6]. Indeed, the probability of cure is highly influenced by the characteristics of the pathogen involved, implying that the identification of pathogens considerably improves mastitis treatment protocols [6].

Bacteria causing clinical mastitis may be contagious or environmental in origin [1] and for this the disease is categorized as contagious or environmental. The bacteria associated with either form in industrialized settings are well described [5], [6], [7]. It is documented that contagious mastitis is caused by *Staphylococcus aureus, Streptococcus agalactiae*, and *Streptococcus dysgalactiae* [5], [6], and the udder is the primary reservoir of contagious pathogens. The mode of spread is from the infected quarter(s) to other quarters and cows primarily at milking time. On the other hand, environmental mastitis can be caused by coliforms (*Escherichia coli*, *Klebsiella pneumoniae*, *Klebsiella oxytoca* and *Enterobacter aerogenes*); environmental Streptococci (*Streptococcus uberis*, *Streptococcus bovis* and *Streptococcus dysgalactiae*); and Enterococci (*Enterococcus faecium* and *Enterococcus faecalis*). The environment of the cow is the primary source of infection [1].

The above classification notwithstanding, it is now recognized that the distinction between contagious and environmental mastitis is not always clear and some bacteria can have contagious and environmental modes of transmission. As such, surveillance data has revealed changes in mastitis isolate profiles, which, among other factors, are also influenced by setting [5], [6], [7], [8], [9]. This again emphasizes the need for periodic evaluation of bacteria associated with mastitis. Indeed, until recently coagulase negative staphylococci (CoNS) were considered to be less virulent and mainly associated with subclinical mastitis. Yet, several studies in Europe and North America now reveal that CoNS can cause clinical mastitis [5], [6], [7], [9].

Furthermore, with the global increase in antimicrobial resistance and zoonotic diseases, it has become important to periodically determine profiles and antimicrobial susceptibility patterns of pathogens associated with bovine mastitis. Indeed, the problem of antimicrobial resistance has been blamed in part on the heavy usage of antimicrobials in livestock production. Antimicrobials are routinely used for therapeutic treatment of disease, at sub-therapeutic concentrations to prevent disease (prophylaxis) and for growth promotion [10], [11]. For instance, in Finland, cattle were reported to be the most treated animal species [12] in which clinical mastitis was the most common indication for antimicrobial treatment followed by subclinical mastitis [12].

Clearly, to elucidate mechanisms underlying the alarming global trends in antimicrobial resistance, careful characterization of antimicrobial resistance patterns among bacteria from food animals particularly cattle is paramount. This requires use of reliable methods in obtaining data on the bacterial distribution and defining the profiles of species involved [9], [13], [14]. Moreover, such data is also useful for infection control and in the development of guidelines for appropriate antimicrobial usage in Veterinary Medicine [5], [6], [15] [16].

Through conventional procedures and automated microbial identification system, here we describe the distribution and antimicrobial susceptibility patterns of bacteria associated with clinical bovine mastitis in and around Kampala, Uganda. Bovine samples were from cases reported by farmers for veterinary care**.** Due to the high concern of zoonotic infections, nasal swabs were simultaneously collected from milkmen to compare isolate profiles.

## Results

Over the period of 1 year (February 2010 through March 2011), 97 bovine milk samples from cows with clinical mastitis in Kampala were studied for the distribution of bacterial species and antimicrobial susceptibility patterns. One sample per animal was collected representing a total of 97 cows that were sampled. Most cows belonged to exotic cattle breeds (Holstein Friesian, Jersey, Guernsey; 52%, 50/97) or their crosses with indigenous cattle (43%, 42/97); five (5%, 5/97) belonged to local breeds (East African Zebu and Ankole).

Bovine samples were from a total of 34 farm units; 16 dairy farms (50 samples), 17 zero-grazing units (35 samples) and one communal grazing unit (12 samples), Table S1. Most exotic and cross-breed cows were under organized farm units (dairy farms or zero-grazing) while the indigenous cows were under communal grazing. However, 15 cross-breeds were under the communal grazing scheme (Table S1).

### Identification of Bacteria

Following initial culturing and determination of Gram staining properties, pure cultures were grown from single colonies, and isolates were confirmed to species level through conventional procedures and the Phoenix 100 ID/AST automated system [17], [18], [19], [20]. Due to controversy over the efficiency of this system in identification of Gram-negative bacteria [18], [21], presumptive Gram negatives were identified through conventional methods before subjecting to Phoenix 100 ID/AST. Generally there was agreement between the Phoenix 100 ID/AST system and conventional methods in the identification of common Gram negatives (*Escherichia coli*, Klebsiella and *Proteus vulgaris*). However, isolates of rare organisms, Gram-positive and Gram-negative alike (Lactococci, Micrococci, Arcanobacteria, Cedecea, Serratia, Citrobacter and Leclercia), were identified with Phoenix 100 ID/AST.

### Bacterial Distribution in Milk (Bovine Samples)

Bacteria grew from 82 milk samples (85%, 82/97) of which 49 (51%, 49/97) grew pure cultures. Twenty two (23%, 22/97) samples had mixed cultures but with a predominant colony type which was pursued for further analysis. Eleven (11%, 11/97) samples had mixed growth, from which pure cultures and selection for further analysis depended on medical/veterinary importance judged from morphological features of cells/colonies. Ultimately, one isolate per sample was considered in further analyses.

There was no growth in 11 samples (11%, 11/97) while three (3%, 3/97) were contaminated (at the site of collection) hence discarded; one sample grew *Candida albicans* and was not included in analyses. The bovine samples with no growth and those contaminated on-site were mostly from cows under the communal grazing scheme. This may reflect difficulty encountered in sampling these animals (e.g. lack of restraint facilities to facilitate cleaning of the udder and sampling). Nevertheless, mastitis of viral origin, mycoplasma or un-cultivatable bacterial species may also be responsible for the negative cultures.

i) **Gram-positive bacterial species.** There were 58 isolates of Gram-positive bacteria (58/82, 71%), of which only one was *Staphylococcus aureus* (1/58, 2%) while 20 were coagulase negative Staphylococci (CoNS), (20/58, 34%). CoNS were identified as *Staphylococcus hycus* (4), *Staphylococcus saprophyticus* (4), *Staphylococcus xylosus* (3), *Staphylococcus sciuri* (2), *Staphylococcus epidermidis* (1), *Staphylococcus haemolyticus* (1), *Staphylococcus hominis* (1), *Staphylococcus lugdunensis* (1), *Staphylococcus gallinarum* (1), *Staphylococcus pasteuri* (1) and *Staphylococcus intermedius* (1).

Enterococci were 16 (16/58, 28%) identified as *Enterococcus faecium* (5), *Enterococcus hirae* (4), *Enterococcus faecalis* (3), *Enterococcus gallinarum* (2), *Enterococcus durans* (1) and *Enterococcus raffinosus* (1). Streptococci were 13 (13/58, 22%), identified as *Streptococcus bovis* II (5), *Streptococcus acidominimus* (3), *Streptococcus uberis* (3), *Streptococcus angionosus* (1) and *Streptococcus* group C/G (1). Additionally, Lactococci, Micrococci and Arcanobacteria were detected in eight samples, speciated as; *Lactococcus lactis* species *lactis* (4/58, 7%)*; Lactococcus garvieae* (1/58, 2%)*; Micrococcus lylae* (2/58, 3%), and *Arcanobacterium pyogenes* (1/58, 2%).

ii) **Gram-negative bacteria.** Twenty four (24/82, 29%) coliforms were identified half of which were *Escherichia coli* (12/24, 50%). The others were *Klebsiella oxytoca* (5), *Proteus vulgaris* (2), *Serratia marcescens* (2), *Cedecea davisae* (1), *Citrobacter freundii* (1) and *Leclercia adecarboxylata* (1).

Thus, in Kampala and surrounding areas, CoNS, Enterococci, Streptococci and *Escherichia coli* are the predominant bacteria associated with clinical mastitis. The isolate profiles are summarized in Table S1.

### Bacterial Distribution in Nasal Swabs (Humans)

Thirty one nasal swabs from milkmen grew 24 (24/31, 77%) bacterial isolates with no growth occurring in seven (7/31, 23%); Gram-negative organisms were not detected (not surprising since nares are not conducive for their growth).

Eleven Staphylococci (11/31, 35%) were detected in humans of which four were *Staphylococcus aureus*. This implies that the nasal carriage of *Staphylococcus aureus* in milkmen was 13% (4/31), lower than that reported in hospital settings in Uganda [22]. Additionally, similar species of CoNS to those detected in cows were identified; *Staphylococcus scuiri* (3), *Staphylococcus saprophyticus* (2), *Staphylococcus xylosus* (1) and *Staphylococcus intermedius* (1). Importantly, these CoNS were detected in milkmen working on the same farms where similar CoNS were detected in cows, Table S1.

Also detected in humans were eight Enterococci (8/31, 26%) identified as *Enterococcus faecium* (4), *Enterococcus faecalis* (2) and *Enterococcus hirae* (2). Additionally, two isolates were detected for each of *Streptococcus pneumoniae* and *Streptococcus bovis* II (group D), while one was identified for *Lactococcus lactis* species *lactis*.

Overall, the bacterial species detected in milkmen were similar to those identified in bovine samples, Table S1. While this alluded to a possibility of transmission between humans and cows, largely, genotyping data did not support this notion (see ‘Genotyping’ below).

### Antimicrobial Resistance Patterns

There were high levels of antimicrobial resistance among isolates from cows and milkmen;

i) **Antimicrobial resistance among Staphylococci from cows.** All Staphylococci (21/21, 100%) from bovine samples were susceptible to daptomycin, ciprofloxacin, mupirocin, moxifloxacin, linezolid and gentamicin. However, all the isolates (21/21 100%) were resistant to ampicillin and penicillin G; expectedly, all were found to be “beta-lactamase” producers. Of note, the sole isolate of *Staphylococcus aureus* from cows was susceptible to cefoxitin and oxacillin implying it was methicillin susceptible *S. aureus* (MSSA). However, most isolates of CoNS were resistant to cefoxitin (12/21, 57%) and oxacillin (12/21, 57%), implying they were methicillin resistant Staphylococci (MRS). Furthermore, Staphylococci were substantially resistant to amoxicillin-clavulanate (11/21, 52%), tetracycline (7/21, 33%) and trimethoprim-sulfamethoxazole (6/21, 29%).

Moreover, there were two vancomycin resistant staphylococci (*Staphylococcus hominis* and *Staphylococcus lugdunensis*) which were also MRS (i.e. methicillin-resistant-vancomycin-resistant staphylococci, MR-VRS). Of note, while resistance to rifampicin was low (2/21, 10%), the two MR-VRS isolates were the ones resistant to this drug, Table S2.

ii) **Antimicrobial resistance among Staphylococci from milkmen.** All the 11 staphylococci from humans were susceptible (11/11, 100%) to daptomycin, rifampicin, muprocin, moxifloxacin, linezolid and gentamicin. Thus, the difference in pan-susceptibility between bovine and human isolates was ciprofloxacin to which three human-isolates were resistant (while all from cows were susceptible) and rifampicin, to which two bovine MR-VRS were resistant (while all from humans were susceptible), Tables S1 and S2.

Similar to the antimicrobial susceptibility patterns of bovine isolates, all the 11 (11/11, 100%) human isolates were resistant to ampicillin and penicillin G, and were also found to be “beta-lactamase” producers. Again, the four human isolates of *Staphylococcus aureus* were susceptible to cefoxitine and oxacillin implying they were MSSA. However, the human MSSA were also resistant to trimethoprim-sulfamethoxazole and tetracycline. Furthermore, as with bovine isolates, human CoNS were substantially resistant to cefoxitin (7/11, 64%) and oxacillin (7/11, 64%) implying that they were also MRS. Also, resistance to amoxicillin-clavulanate (7/11, 64%) and tetracycline (7/11, 64%) was substantial.

Furthermore, three MRS (*Staphylococcus scuiri*) resistant to vancomycin were detected in humans, Tables S1 and S2.

While the species distribution between humans and cows was similar (i.e. MRS in milkmen -*Staphylococcus sciuiri*, *Staphylococcus saprophyticus*, *Staphylococcus xylosus* and *Staphylococcus intermedius* were also detected in cows), the antimicrobial resistance patterns differed. Furthermore, while all the vancomycin resistant staphylococci (VRS) from humans and bovines were MRS, the species were different (i.e. VRS from cows were *Staphylococcus hominis* and *Staphylococcus lugdunensis* while the one from milkmen was *Staphylococcus sciuiri*). Overall, the staphylococcal species from bovine samples that were not detected in milkmen were *Staphylococcus hycus*, *Staphylococcus lugdunensis* and *Staphylococcus gallinarum*.

**Figure 1:**
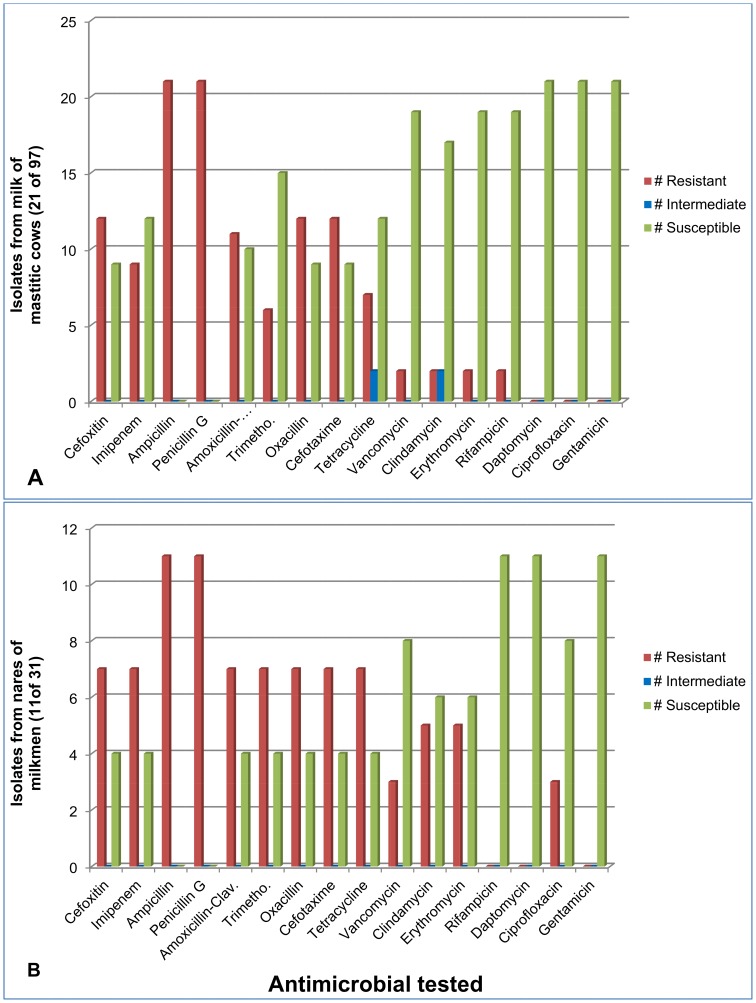
Antimicrobial resistance among staphylococci from cows (panel A) and milkmen (panel B). Details in Tables S1 and S2.

Figure 1 graphically depicts the antimicrobial resistance among staphylococci in cows and milkmen. Details for the susceptibility patterns of each isolate are provided in Table S2.

iii) **Antimicrobial resistance among Enterococci from cows.** All Enterococci (16/16, 100%) from bovine samples were susceptible to ampicillin. However, resistance to tetracycline (5/16, 31%), vancomycin (3/16, 19%), teicoplanin (13%, 2/16), erythromycin (3/16, 19%), daptomycin (1/16, 6%), and ciprofloxacin (1/16, 6%) was noted, but relatively low, Table 1.

**Table 1:** Antimicrobial resistance patterns among Enterococci.

Isolates from cows (n = 16)		Comment
Species/isolate	Antimicrobial resistance pattern	
*E. faecium*	ERY	
***E. faecium***	DAP-ERY	DRE
*E. faecium*		
*E. faecium*		
*E. faecium*	ERY	
*E. faecalis*	ERY-CIP-TET	
*E. faecalis*	ERY-TET	
***E. faecalis***	TEI-VAN	VRE
*E. hirae*	ERY-TET	
*E. hirae*		
*E. hirae*	TET	
*E. hirae*	ERY	
***E. gallinarum***	TEI-VAN	VRE
***E. gallinarum***	TEI-VAN	VRE
*E. durans*	TET	
*E. raffinosus*	ERY-TET	
Isolates from humans (n = 8)		
*E. faecalis*	ERY-TET	
*E. faecalis*	ERY-CIP-TET	
*E. faecium*	TET	
*E. faecium*		
*E. faecium*	ERY	
*E. faecium*		
*E. hirae*	ERY	
*E. hirae*	TET	

DAP, Daptomycin; TEI, Teicoplanin; VAN; Vancomycin; ERY, Erythromycin; CIP, Ciprofloxacin; TET, tetracycline.

In boldface type are isolates that were found to be resistant to daptomycin and vancomycin, respectively (i.e., DRE, daptomycin resistant enterococcus, and VRE, vancomycin resistant enterococcus).

Of concern was the detection of isolates resistant to vancomycin and daptomycin (*Enterococcus faecalis* and *Enterococcus gallinarum*, respectively), Table 1. Since these drugs are crucial in the treatment of infections due to intractable pathogens, detection of such isolates in milk is risky to consumers in case it is consumed raw.

iv) **Antimicrobial resistance among Enterococci from milkmen.** All enterococci (8/8, 100%) from humans were susceptible to ampicillin, daptomycin, teicoplanin, vancomycin and moxifloxacin while resistance to erythromycin was also low (13%, 1/8), Table 1.

Lastly for the Gram-positives, Streptococci, Lactococci, Micrococci and Arcanobacteria were also identified to species level but the susceptibility patterns for these organisms are not included in the Phoenix 100 AST panels hence are not reported.

v) **Antimicrobial resistance among Gram-negative isolates.** All coliforms were susceptible to amikacin, gentamicin, imipenem, meropenem, ceftazidime, ciprofloxacin and levofloxacin, Table 2. However, resistance to ampicillin was high (17/24, 71%) while it was moderate for cephalothin (8/24, 33%), trimethoprim-sulfamethoxazole (8/24, 33%), cefuroxime (6/24, 25%) and amoxicillin-clavulanate (5/24, 21%). Also, resistance to nitrofurontoin (4/24, 17%), colstin (4/24, 17%), cefoxitin (1/24, 4%), ertapenem (1/24, 4%), cefepime (1/24, 4%), aztreonam (1/24, 4%) and piperacillin (1/24, 4%) was low. Although coliforms were the least resistant with three pan-susceptible isolates, multidrug resistant (MDR) isolates (*Klebsiella oxytoca*, *Proteus vulgaris*, *Serratia marcescenes, Cedecea davisae,* and *Citrobacter freundii*) were detected, Table 2.

**Table 2:** Antimicrobial resistance patterns among coliforms (n = 24).

Species	Antimicrobial resistance pattern	Comment
***Citrobacter freundii***	AMP-AMO-CEF-CEP-CFU	MDR
*Escherichia coli*	CEP	
*Escherichia coli*	CEP	
*Escherichia coli*	CEP	
*Escherichia coli*	AMP-AMO-SXT-CEP	
*Escherichia coli*	–	Pan-susceptible
*Escherichia coli*	AMP-CEP	
*Escherichia coli*	AMP-SXT-CEP	
*Escherichia coli*	AMP-SXT-CEP	
*Escherichia coli*	AMP-SXT	
*Escherichia coli*	CEP	
*Escherichia coli*	AMP-AMO-CEP	
*Escherichia coli*	AMP	
*Klebsiella oxytoca*	SXT	
*Klebsiella oxytoca*	–	Pan-susceptible
***Klebsiella oxytoca***	SXT-CEF-CEP-CFU-CFP-AZT-PIP	MDR
*Klebsiella oxytoca*	SXT	
***Klebsiella oxytoca***	SXT-CEF-CEP-CFU-CFP-AZT-PIP	MDR
*Leclercia adecarboxylata*	–	Pan-susceptible
***Proteus vulgaris***	AMP-SXT-COL-CEP-CFU-NTR	MDR
*Proteus vulgaris*	AMP-NTR	
***Serratia marcescenes***	AMP-AMO-COL-CEP-CFU-NTR	MDR
***Serratia marcescenes***	AMP-AMO-COL-CEF-CEP-CFU-NTR	MDR
*Cedecea davisae*	AMP-AMO-COL-CEF-CFT-CEP-CFU-NTR-ERT	MDR

AMP, Ampicillin; AMO; Amoxicillin-Clavulanate; SXT, trimethopprim-sulfamethoxazole; COL, Colistin; IMP, imipenem; CEF, Cefoxitine; CFT, Cefotaxim; CEP, Cephalothin; CFU, Cefuroxime; CFP, Cefepime; AZT, Aztreonam; ERY, Erythromycin; NTR, Nitrofurantoin; PIP, Piperacillin-Tazobactum; ERT, Ertapenem.

In boldface type are isolates found to be multi-drug resistant (MDR).

### Genetic Relatedness among Human and Bovine Isolates

The similar bacterial species that were detected in milkmen and cows (Staphylococci; Enterococcus; Streptococcus; Micrococcus) were genotyped to determine relatedness and possible transmission between humans and livestock. *Staphylococcus aureus* was genotyped with spa typing while multi-locus sequence typing (MLST) was performed for the two Enterococci that were resistant to vancomycin and daptomycin. However, owing to the diversity of the species involved and paucity of genotyping methods, as well as cost implications, RAPD genotyping was employed for the other isolates.

Following MLST, the vancomycin resistant *Enterococcus faecalis* was found to be unique with an allelic profile of 1 (*gdh*); 1 (*gyd*); 3 (*pstS*); 7 (*gki*); 21 (*aroE*); 1 (*xpt*); 5 (*yqil*); close to *E. faecalis* ST447 [allelic profile of 1 (*gdh*); 7 (*gyd*); 3 (*pstS*); 7 (*gki*); 6 (*aroE*); 1 (*xpt*); 5 (*yqil*)]. This strain was submitted to the MLST database to assign the sequence type. However, the daptomycin resistant isolate of *Enterococcus faecium* was found to be un-typable.

**Figure 2:**
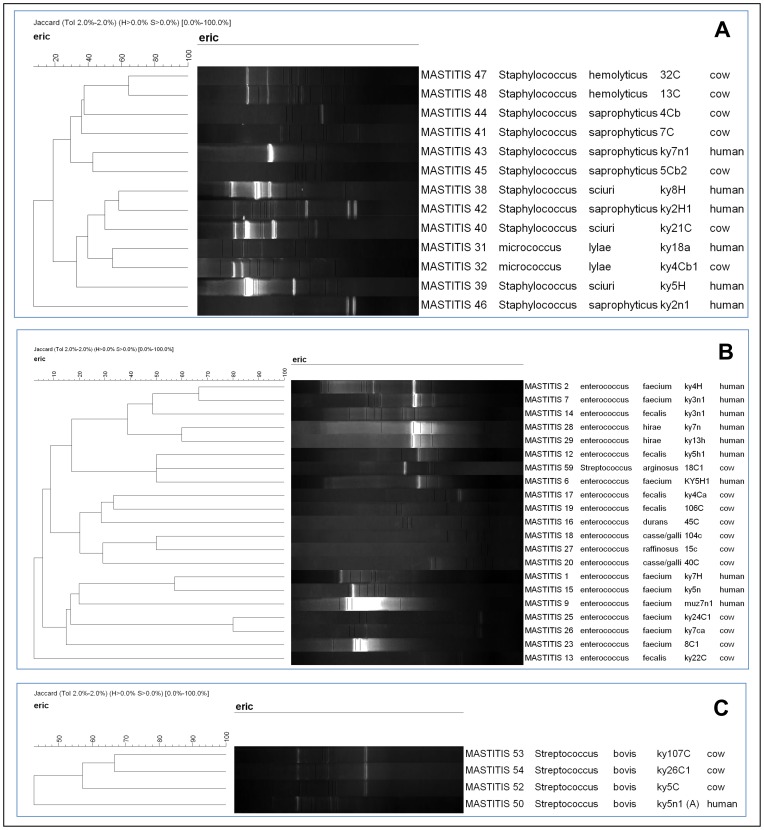
Distinct patterns among staphylococci (panel A), enterococci (panel B) and streptococci (panel C) following RAPD genotyping. One isolate per lane.

Overall, while similar bacterial species were detected in human and bovine samples, and often on the same farm (Table S1), the genotyped isolates displayed distinct patterns, Figure 2. Thus, transmission between milkmen and cows was not detected (at least for these isolates).

Interestingly however, isolates of *Staphylococcus aureus* were genetically similar; all strains, human and bovine alike, belonged to the same lineage, spa type t645 (spa-CC435, ST121) implying genetic relatedness, Table 3. Moreover, the bovine and human isolates were detected in samples from the same farm, Table 3. However, as described above, the drug susceptibility data for the bovine isolate was different from that of human isolates in that the latter were resistant to trimethoprim-sulfermethaxole (SXT) and tetracycline (in addition to ampicillin and penicillin G to which the bovine isolate was resistant). In an attempt to account for this difference, we performed plasmid profiling and indeed identified differences among bovine and human isolates. All isolates possessed an approx. 20 kb plasmid; however, the human isolates were found to possess three additional smaller plasmids (approx. 5, 4 and 3 kb, respectively) that were missing in the bovine isolate. Since antimicrobial resistance genes including those encoding SXT and tetracycline resistance are plasmid-encoded, the difference in susceptibility patterns may be attributed to the acquisition of plasmids by the human isolates.

**Table 3:** *Staphylococcus aureus* from cows (n = 1) and humans (n = 4) with similar Spa type, t645.

Date of collection	Isolate number	Source	Location	DST^a^	Spa repeat	Spa type	ST
23-Feb-2010	Ky9c	Cow (milk)	Katale (Farm A1)	RRSSSSSSSSSSSS	14∶44∶13∶12∶17∶23∶18∶17	t645	ST-121
23-Feb-2010	Ky17n	Human (nares)	Katale (Farm A1)	RRRRSSSSSSSSSS	14∶44∶13∶12∶17∶23∶18∶17	t645	ST-121
3-Jul-2010	Ky2n	Human (nares)	Kisubi (Farm A9)	RRRRSSSSSSSSSS	14∶44∶13∶12∶17∶23∶18∶17	t645	ST-121
10-Dec-2010	Ky6n	Human (nares)	Entebbe (Farm B1)	RRRRSSSSSSSSSS	14∶44∶13∶12∶17∶23∶18∶17	t645	ST-121
4-Mar-2011	105n	Human (nares)	Wakiso (Farm A12)	RRRRSSSSSSSSSS	14∶44∶13∶12∶17∶23∶18∶17	t645	ST-121

a Drug susceptibility testing. R, Resistant, S, susceptible, with respect to drugs in the following order: Ampicillin; Penicillin G; Trimethopprim-sulfamethoxazole; Tetracycline; Cefoxitine; Oxacillin; Amoxicillin-Clavulanate; Teicoplanin; Vancomycin; Clindamycin; Erythromycin; Nitrofurantoin; Rifampicin; Ciprofloxacin.

All *S. aureus* were methicillin susceptible (MSSA).

Nevertheless, the different DST patterns negate the possibility of transmission in spite of the isolates being genetically similar.

### Highlight on Management Practices

For an insight into the management practices among the farm units where samples were collected, a formal survey focusing on veterinary care and milking practices was conducted using an interview administered questionnaire;

### Veterinary Care and Antimicrobial Usage

Farmers with dairy farms and zero grazing units reported that they relied on veterinarians for veterinary services whenever they encountered clinical mastitis. However, farmers practicing communal grazing relied on milkmen and herdsmen to treat mastitis and involved veterinarians only when they encountered difficulty. Intramammary infusions with ampicillin or tetracycline were frequently used by most farmers. Also occasionally used were anti-inflammatories such as calvasone, predinisolone, and dexamethathone.

Furthermore, most farmers reported poor response to treatment particularly with ampicillin-based intramammary infusions (which may be explained by the high proportion of isolates resistant to this drug found in this study). Indeed, MRS (*Staphylococcus hycus*) isolates were recovered in four cases where farmers reported poor response to treatment. The intramammary infusions used to treat these cows contained penicillin to which all staphylococci were resistant. Following drug susceptibility testing (DST), gentamicin-based infusions were advised and a good response was reported (i.e. cows were cured of clinical mastitis). However, the recommended withdraw period was not observed in that farmers continued to consume or sell the milk from cows on treatment.

### Milking Practices and Udder Hygiene

Milking machines are rare in this setting and most farmers rely on hand-milking. Nevertheless, the milking technique employed by milkmen was poor (i.e. pulling teats instead of squeezing them). For dairy farms, there was no specific order of milking cows with respect to health status (e.g. milking healthy cows before sick ones). Teat dipping was practiced only on one farm. Furthermore, communal grazing-farmers used the same individual (a herdsman) for milking cows from different herds as he gathered cattle for grazing.

Taken together and considering the isolate profiles described, clinical mastitis in this setting is mostly environmental [1].

## Discussion

In this study, we have employed contemporary bacterial identification procedures to describe the bacterial species associated with clinical mastitis in Kampala, Uganda. Isolates which previous studies in Uganda could not identify [2], [3] have been elucidated through the use of the Phoenix 100 ID/AST automated system. Staphylococci, Enterococci and Streptococci from milkmen and livestock were identified to species level, as well as rare organisms such as Micrococcus, Arcanobacteria, Cedecea, Serratia, Citrobacter and Leclercia. Overall, CoNS, Enterococci, Streptococci and *Escherichia coli* were the predominant bacteria associated with clinical mastitis in Kampala. These organisms are notorious agents of mastitis globally particularly in Europe [8], [23] and Asia [24] [25].

While further studies may be required, one can assume that in Kampala, environmental clinical mastitis, for which coliforms are most incriminated [1], is prevalent and may surpass the contagious form of disease. This may not be surprising given the low levels of hygiene and inappropriate husbandry practices encountered in this study. Environmental mastitis usually reflects poor management practices [1], as previously reported [3]. Nevertheless (and given the ambiguous understanding of mastitis disease forms), contagious mastitis, also usually due to poor management practices particularly at milking [1], could as well have contributed to the high prevalence of environmental pathogens detected.

Subclinical mastitis has been studied before in Uganda [2], [3]; however, there’s scanty data on clinical mastitis. Therefore, any comparison with previous studies in Kampala and Uganda in general is with respect to subclinical mastitis. In a previous study [3], penicillin and oxacillin resistance was reported to be 86.8% and 29.7%, respectively, while in the current study it was 100% and 57%, respectively [3]. Furthermore, resistance to tetracycline in the previous study was higher than what we have reported (86% vs. 33%). It is postulated that penicillin and tetracycline resistance is exacerbated by the frequent usage by farmers of intramammary infusions with those drugs [3]. Also in the previous study resistance to gentamicin was reported albeit low while it was not detected in the current study. The low gentamicin resistance in Uganda has been attributed to the high cost of this drug which prohibits its usage by farmers [3], in the end slowing emergence of resistance.

Furthermore, in a recent report on subclinical mastitis in peri-urban Kampala [2], infection with CoNS (54.7%) and Streptococci (16.2%) was found to be the most common bacteriological outcome [2]. Six of the nine (67%) CoNS and four of the eight (50%) *Staphylococcus aureus* were positive for penicillinase production. Although substantially high, this contrasted with the absolute (100%) beta-lactamase production among Staphylococci in the current study; the disagreement could be attributed to differences in methodology. Interestingly however, the prevalence of *Staphylococcus aureus* (an organism highly associated with bovine mastitis) was very low in both studies (i.e. of the 450 quarter samples in the former study, *Staphylococcus aureus* grew only in eight while CoNS grew in 246) [2].

### Transmission of Bacteria between Milkmen and Cows was not Detected

While transmission of bacterial species between humans and livestock is increasingly being detected in farm workers in Europe and much of the industrialized world [26], there’s so far no report to indicate the same occurs in sub-Saharan Africa. Moreover, methicillin resistant *Staphylococcus aureus* (MRSA), a common finding in livestock workers [5], was not detected in this study. Of concern however was the detection in cows and milkmen of high levels of MDR bacteria of the same species implying that transmission is possible. For most species however, transmission was not detected in that the human and bovine isolates displayed unrelated DST and RAPD patterns, implying that they were indeed different.

However, the exception was *Staphylococcus aureus* for which a bovine isolate presented a similar spa type to that of humans’. Interestingly, the bovine and a human isolates were collected on the same farm. Yet, the different DST patterns among these isolates negate occurrence of transmission between milkmen and cows.

Resistance genes in *Staphylococcus aureus* are often plasmid-encoded and disseminate through *Staphylococcus aureus* populations by horizontal gene transfer (HGT) mechanisms leading to strains that are more resistant [27], [28], [29]. Thus, it’s possible that the plasmids detected in the human isolates were acquired through HGT and encode resistance to SXT and tetracycline. Of note, the identified strain belonged to a lineage that occurs worldwide [30], spa Type t645 (spa-CC435, ST121), and it was also the most predominant lineage among *Staphylococcus aureus* causing surgical site infections [31] at Mulago hospital, a national referral hospital in Kampala. To date, there’re five strains of lineage t645 in the Ridom database [http://spa.ridom.de/spa-t645.shtml] associated with infection.

### Situation in the Rest of Africa

Generally there’s little data on bovine mastitis from sub-Saharan Africa. Nevertheless, we highlight our findings in light of countries where mastitis has been documented irrespective of disease form. Since climate and management practices markedly differ between countries, we only compare isolate profiles without accounting for differences.

In an Algerian study the majority of bacteria from cows with subclinical mastitis were CoNS [32]; another similar finding is that *Lactococcus lactis* species *lactis* was also isolated [32]. However, there was higher susceptibility of the isolates to antimicrobials including penicillin, contrary to the findings in this study. While one may point to differences in enforcement of regulations on antimicrobial usage between Uganda and Algeria, MDR-CoNS are prevalent in Nordic countries notable for sound antimicrobial regulations [33].

Meanwhile in the Sudan, Staphylococci also dominated isolates recovered from cows with clinical and subclinical mastitis [34]. Interestingly, *Arcanobacterium pyogenes,* an emerging etiological agent for bovine mastitis [35], was also identified in the Sudanese study.

Overall, the bacterial distribution in Africa appears similar but with some important exceptions. For instance, there are differences in antimicrobial susceptibility patterns between isolates reported in our study and those from Algeria; the isolate distribution also differs between our study and the Sudanese (i.e. Corynebacteria, Brucella, Pseudomonas and Aerococcus were detected in Sudan but not in the current study).

### Europe

Given the contrast in animal husbandry practices and in enforcement of antimicrobial usage between Uganda and Europe, this discussion only serves to highlight global trends in isolate profiles and antimicrobial resistance patterns without accounting for differences or similarities.

In Europe, there are varying reports both in the distribution and antimicrobial susceptibility patterns of bacteria causing mastitis. For instance in Finland, CoNS dominated isolates from cows with clinical mastitis in which symptoms were most severe in cows with *Staphylococcus hycus* infection [33]. Interestingly, in the current study, *Staphylococcus hycus* was also among the most prevalent among the CoNS. Meanwhile in Estonia, the main bacterial pathogens associated with clinical mastitis were *Streptococcus uberis* and *Escherichia coli* [36] while subclinical mastitis was caused mainly by *Staphylococcus aureus* and CoNS. Similar to our findings, antimicrobial resistance was prevalent in Estonia, especially penicillin resistance among *Staphylococcus aureus* and CoNS. In Switzerland, high prevalence of MRS was found in livestock production facilities [37] and in addition to beta-lactam resistance, most strains were resistant to other non-beta-lactam antibiotics [37]. Yet in Sweden, *Staphylococcus aureus* and CoNS are frequently associated with subclinical mastitis but antimicrobial resistance is very low [21].

Of note, human-CoNS species tend to be MDR yet their counterpart, *Staphylococcus aureus*, is less prone to developing multi-resistance to antimicrobials particularly in the Nordic countries [15] [16]. Also, CoNS species from bovines in Europe are most of the time reported to be susceptible to antimicrobials [9], [13], [14], in contrast with CoNS in this study. Differences in animal husbandry, management practices as well as enforcement of antimicrobial regulations could account for this. In veterinary medicine, CoNS have become a problem and are currently incriminated as causes in several episodes of clinical mastitis.

### Limitations

There’re some shortcomings in this report. First, the study was based on mastitis cases from Kampala reported by farmers and represents only those who could afford veterinary care. Thus, these findings are not generalizable to the entire city or country. Also, some animals were on medication and this could have affected recovery of bacterial isolates. Additionally, most milkmen didn’t consent limiting the human-sample size.

Secondly, whilst utmost care was taken to minimize contamination through strict adherence to standardized sampling procedures, it is possible that some isolates could have been contaminants from the cows’ environment given the ubiquity of bacteria on cows. Nevertheless, the observed improvement in cure rates among stubborn cases following DST implies that contamination was really minimal. Also, there was no bacterial growth in several samples, bovine and human alike. Moreover, even in settings with developed dairy industries, bacterial species previously thought to be commensals or contaminants are now documented causes of clinical mastitis [13], [14]. It is increasingly becoming clear that there may be no difference between microbes formerly considered pathogenic vs. the nonpathogenic ones [http://www.einstein.yu.edu/uploadedFiles/casadevall/10_Casadevall_Pirofski_09.pdf].

## Conclusions

Bovine clinical mastitis mainly due to CoNS, Enterococci, Streptococci and *Escherichia coli* is prevalent in Kampala, Uganda. Multidrug resistant bacteria notably coagulase negative Staphylococci and coliforms other than *Escherichia coli* (*Klebsiella, Proteus, Serratia, Citrobacter* and *Cedecea*) are also prevalent. Of concern was the detection of vancomycin and daptomycin resistant Enterococci in cows, as well as methicillin and vancomycin resistant staphylococci both in milkmen and cows. While the potential for transmission of bacteria between humans and livestock occurs, it was not detected in this study given the different genotypic and susceptibility patterns exhibited by the isolates. Further studies are required to ascertain this.

## Materials and Methods

### Ethics Statement

Written informed consent was sought from all the milkmen who participated and those who did not consent were excluded. Additionally, the study protocol and consent procedure were approved by the Uganda National Council of Science and Technology (UNCST) (reference # NS 371). The UNCST registers and clears all research intended to be carried out in Uganda and in so doing, it reviews the research protocols for their scientific merit, safety and ethical appropriateness prior to issuing permits for conducting studies. The research permit is granted at a national level to facilitate the carrying out of research within the country. All research in Uganda is registered and approved by the UNCST [38].

### Setting

This study was conducted within farming units of Kampala and surrounding areas including the adjoining districts of Wakiso, Mukono, Mpigi, Luwero, Kamuli, Kayunga and Mityana [39].

### Definition of Clinical Mastitis

A textbook definition of clinical mastitis was considered [1]; a cow with visible signs of mastitis, either, mild (flakes or clots in milk, slight swelling of infected quarter) or severe (abnormal secretion, hot, swollen quarter or udder, fever, rapid pulse, loss of appetite, dehydration and depression) [1]. As expected, cows with severe signs were more common (since most were cases reported by farmers for veterinary care). Cows were clinically re-examined by field veterinarians to confirm symptoms prior to sample collection.

### Collection of Milk Samples

Information on clinical mastitis cases was obtained from field veterinarians who informed research assistants through telephone calls and a farm visit was arranged. Milk samples were collected consecutively from affected quarter(s) using sterile 50 ml centrifuge tubes (Fisher Scientific, Leicestershire, UK). To minimize contamination, we strictly adhered to the mastitis sample collection protocol described by Dr. J.W. Schroeder, North Dakota State University [www.ag.ndsu.edu/pubs/ansci/dairy/as1129.pdf] [1]. Briefly, centrifuge tubes were labeled and forms filled prior to each farm visit. At the farm, hands were washed with soapy water while teats were washed with 70% ethanol and dried individually with clean paper towels. Two squirts of milk were discarded from the teat before dipping in a germicidal teat dip (which contained 0.64% Sodium Chlorite) for 30 sec of contact time. After wiping off the teat dip with an individual clean towel, the teat end was thoroughly scrubbed with a cotton swab soaked in 70% ethanol. A clean swab was used for each teat. Then, a centrifuge tube was opened under the teat and held at an angle so that foreign material could not fall into the opening; nothing was allowed to come in contact with the mouth of the tube. Approx. 5 ml of milk was collected from each infected quarter, and the container was closed before removing it from beneath the teats. During farm visits, samples were stored briefly in an ice-cold box and promptly transported to the bacteriology laboratory for culture.

### Human Samples

Nasal samples (swabs) were simultaneously collected from milkmen who gave written informed consent, and similarly transported in a separate ice-cold box to the bacteriology laboratory.

### Questionnaire

A formal survey with an interviewer administered questionnaire was conducted to collect data on location, herd size, farming system, clinical symptoms, breed, parity, age, milk-yield, stage of lactation, treatment record and antimicrobial usage. This survey was conducted among farm owners and had a high response rate (100%).

### Bacterial Cultures

Initially, samples were cultured on blood agar or on tryptic soy agar (TSA) (for samples with no growth on blood agar plates). Plates were incubated at 37°C for 24h. Further processing followed the laboratory’s standard operating procedures for identifying Gram-positive and Gram-negative bacteria.

Staphylococci were presumptively identified with a previously described protocol that involves sequel testing of catalase positive isolates with tube coagulase, Mannitol salt agar and DNase tests [40]. *Staphylococcus epidermidis* was confirmed through culturing CoNS isolates on TSA with 20 µg/ml of novobiocin (Sigma-Aldrich, St. Louis, MO, USA). Enterococci were presumptively identified on the basis of catalase-negative, Gram-positive cocci growing in the presence of 40% bile (bile-esculin agar, Difco, Detroit, USA) and on 6.5% NaCl in brain heart infusion (BHI) agar (Oxoid, London, UK) [41]. To distinguish Streptococci from Enterococci, growth in BHI broth with 6.5% NaCl was employed in which case Streptococci did not grow while Enterococci grew.

To isolate Gram-negative bacteria, a sample was plated on TSA, blood and MacConkey agar, and incubated overnight at 37°C for 24h. Pure cultures were obtained by re-streaking single colonies from MacConkey plates on TSA and incubating at 37°C for 24h. Morphological features of isolates on TSA, blood and MacConkey agar were examined prior to a series of biochemical tests for identification of *Escherichia coli*, *Proteus* and *Klebsiella* species. Tests involved sugar fermentation (sucrose, glucose, lactose, triple sugar iron, mannitol); motility (Sulphur Indole & Motility test on ‘SIM’ medium); gas production; oxidase; and utilization of citrate and urea [42].

### Confirmation of Isolates to Species Level and Antimicrobial Susceptibility Testing

To confirm the isolates to species level and their antimicrobial susceptibility patterns, we employed the ‘Phoenix Automated Microbiology System’ (Phoenix 100 ID/AST system) from Becton and Dickson (Franklin Lakes, NJ, USA) [19]. This system has combination testing panels that include: a) identification (ID) side with dried substrates for bacterial identification; b) an antimicrobial susceptibility testing (AST) side with varying concentrations of antimicrobial agents; and c) growth and fluorescent controls at appropriate well locations.

The ID portion of the Phoenix panels utilizes a series of conventional, chromogenic, and fluorogenic biochemical tests to determine the identification of the organism. Acid production is indicated by a change in the phenol red indicator when an isolate is able to utilize a carbohydrate substrate. Chromogenic substrates produce a yellow color upon enzymatic hydrolysis of either p-nitrophenyl or p-nitroanilide compounds. Enzymatic hydrolysis of fluorogenic substrates results in the release of a fluorescent coumarin derivative. Organisms that utilize a specific carbon source reduce the resazurin-based indicator. In addition, there are other tests that detect the ability of an organism to hydrolyze, degrade, reduce, or otherwise utilize a substrate.

Specimen processing and Gram staining procedure was performed according to the manufacturer’s guidelines [19]. Then, Phoenix panels were inoculated with a standardized inoculum according to the manufacturer’s guidelines; occasionally, minor modifications were done as described elsewhere [17], [18], [19]. Briefly, after determining the Gram staining properties of the isolates, nonselective media (blood agar or TSA) was used to prepare fresh pure cultures for isolate ID and AST [19]. Isolates were inoculated into appropriate ID/AST combination panels (Phoenix™ PMIC/ID for Gram-positive and Phoenix™ NMIC/ID for Gram-negative isolates) that were loaded into the instrument and incubated at 35°C, according to the manufacturer’s guidelines. The ID broth was inoculated with bacterial colonies adjusted to a 0.5 McFarland standard. The suspension was poured into the ID side of the Phoenix panel after an aliquot (30 µl) was removed and saved for AST.

For AST, the Phoenix AST Indicator Solution was added to the AST broth tubes and mixed by inversion. The AST side of the combination panel contains 84 wells with dried antimicrobial panels and one growth control well [17]. One free-falling drop of the AST indicator was added to the AST broth tube [17], and 30 µl of the standardized ID broth suspension was transferred to the AST broth and incubated up to 16 hours at 35 °C. Samples were read automatically at the instrument’s set parameters.

Quality control and maintenance were performed according to the manufacturer’s recommendations [17]. *Staphylococcus aureus* ATCC^™^ 29213 and *Enterococcus faecalis* ATCC^™^ 29212 were included in the ID and AST Panels for quality control.

### Genotyping

To determine genetic relatedness and whether transmission of bacteria occurs between humans and livestock, genotyping was performed on isolates of the same species that were detected in milkmen and cows.

i) ***Staphylococcus aureus.*** The x-region of *Staphylococcus aureus spa* gene (0.2 kb to 0.4 kb) was amplified by PCR with the method established before [43] using primers 1095F, 5′-AGACGATCCTTCGGTGAG-3′, and 1517R, 5′-CAGCAGTAGTGCCGTTTG-3′. The PCR conditions were as follows: 94 °C for 5 min, followed by 31 cycles each consisting of 94 °C, 30 sec; 53 °C, 30 sec; 72 °C, 1 min and a final extension at 72 °C for 10 min. The PCR products were purified with the QIAquick PCR purification kit (Qiagen, Hilden, Germany) as per the manufacturer’s instructions, and both strands sequenced (ACGT, Wheeling, IL, USA) using the same primers. To obtain *spa* types, the sequences were submitted to a free spaTyper data base (http://fortinbras.us/cgi-bin/spaTyper/spaTyper.pl) and lineages matching to query sequences determined. The data was also submitted to the Ridom Spa server (http://spa.ridom.de/) for comparison.

ii) **CoNS, Enterococci, Lactococci and Streptococci.** The bacterial species belonging to the above genera were genotyped with random amplification of polymorphic DNA (RAPD) typing according to Reinoso et al, 2004 [44], with minor modifications. The primer sequence used was 5′-ACGCAGGCAC-3′, under the conditions: 94°C, 4 min, followed by 40 cycles of 94°C, 1 min, 36°C, 1 min and 72°C, 2 min, with a final amplification step at 72°C for 10 min. Amplicons were analyzed by agarose gel electrophoresis at 90V for 5 hours on a 1% agarose gel. Images were captured with a bioimager and analyzed with the BioNumerics software v. 5 (Applied Maths NV, Sint-Martens-Latem, Belgium).

iii) **Daptomycin and vancomycin resistant Enterococci.** Since daptomycin and vancomycin are important drugs in the treatment of microbial infections, the two Enterococci resistant to these drugs (*E. faecium* and *E. faecalis*, respectively) were typed with multi locus sequence typing (MLST) to ascertain their sequence types. The primers used are summarized in Table S3 and were obtained from [http://www.mlst.net/databases/default.asp].

For *Enterococcus faecium* the following conditions were used; PCR reactions were performed in 50 µl mixture each containing 25 µL HotStar Taq Master Mix (Qiagen), 40 pmol of each primer, and milli-Q water to a final volume of 50 µL. One µl of crude DNA prep was used as template for amplifications. The PCR programme comprised of an initial denaturation at 95°C for 15 min, 35 cycles of 30 s at 94°C, 30 s at 50°C, and 30 s at 72°C, followed by 5 min 72°C. The PCR products were purified with the Qiaquick PCR purification kit following the manufacturer’s instructions, and sequenced at ACGT (Wheeling, IL, USA) with both the forward and reverse primers. Sequence chromatograms were analyzed with BioEdit software and submitted to the MLST database [http://www.mlst.net/databases/default.asp] for sequence types.

For *Enterococcus faecalis* the following conditions were used; initial denaturation at 94°C for 5 min; 30 cycles at 94°C for 30 s, 52°C for 30 s and 72°C for 1 min; and extension at 72°C for 7 min. Reactions were performed in 10 µl volumes with Custom master mix (ThermoFisher, Surry, UK) and Taq polymerase (Thermo-Fisher, Surry, UK). The PCR products were purified as described above for *E. faecium*, sequenced and analyzed similarly.

### Data Analysis

The data was analyzed with STATA SE software version 11.2 (STATA Corp LP, College station TX 77849, USA). A P-value of <0.05 was considered statistically significant.

The gel images for RAPD genotyping data were analyzed with the Bionumerix software (Applied Maths NV, Sint-Martens-Latem, Belgium). The spa and MSLT sequences were analyzed with the BioEdit software and submitted to online databases [http://spa.ridom.de/] and [http://www.mlst.net/databases/default.asp], respectively, to obtain lineages.

## Supporting Information

Table S1 **Isolate profiles (bovine and human samples).** **R,** resistant; **S,** susceptible, with respect to: **Staphylococci:** Ampicillin; Penicillin G; Trimethopprim-sulfamethoxazole; Tetracycline; Cefoxitine; Oxacillin; Amoxicillin-Clavulanate; Teicoplanin; Vancomycin; Clindamycin; Erythromycin; Nitrofurantoin; Rifampicin **Enterococci:** Daptomycin; Teicoplanin; Vancomycin; Erythromycin; Ciprofloxacin; Tetracycline. **Gram-negatives:** Ampicillin; Amoxicillin-Clavulanate; Trimethopprim-sulfamethoxazole; Colistin; Imipenem; Cefoxitine; Cefotaxim; Cephalothin; Cefuroxime; Cefepime; Aztreonam; Erythromycin; Nitrofurantoin; Piperacillin-Tazobactum; Ertapenem NA: Not applicable DRE: Daptomycin resistant enterococcus VRE: Vancomycin resistant enterococcus MSSA: Methicillin susceptible *Staphylococcus aureus* MRS: Methicillin resistant *Staphylococcus* MR-VRS: Methicillin-resistant, vancomycin-resistant *Staphylococcus* DST: Drug susceptibility testing *Zero grazing is an approach to animal management in which families contain livestock in an enclosed, shaded area and carry fodder and water to them instead of letting them wander in the open where they are more likely to catch diseases or damage the environment [http://www.heifer.org.za/faq/what_is_zero_grazing].
(XLS)

Table S2 **Antimicrobial resistance patterns of each staphylococcal isolate.**
(PDF)

Table S3 **Primers for genotyping daptomycin and vancomycin resistant enterococci.**
(PDF)

## References

[1] Schroeder J (2012) Bovine Mastitis and Milking Management. North Dakota State University. Available: www.ag.ndsu.edu/pubs/ansci/dairy/as1129.pdf. Accessed 2013 March.

[2] Abrahmsén M (2012) Prevalence of Subclinical Mastitis in Dairy Farms in Urban and Peri-urban Areas of Kampala, Uganda. Swedish University of Agricultural Sciences. Available: stud.epsilon.slu.se/4213/1/Abrahmsén_m_120507.pdf. Accessed 2012 December. 10.1007/s11250-013-0455-7 PMC3895220 23955012

[3] Byarugaba DK, Nakavuma J, Vaarst M, Laker C (2008) Mastitis occurrence and constraints to mastitis control in small holder dairy systems in Uganda. Livestock Research for Rural Development 20. Available: www.lrrd.org/lrrd20/1/byar20005.htm. Accessed 2013 March.

[4] Hagnestam-Nielsen C, Ostergaard S (2009) Economic impact of clinical mastitis in a dairy herd assessed by stochastic simulation using different methods to model yield losses. Animal 3: 315–328. 22444235 10.1017/S1751731108003352

[5] Bengtsson B, Unnerstad HE, Ekman T, Artursson K, Nilsson-Ost M, et al (2009) Antimicrobial susceptibility of udder pathogens from cases of acute clinical mastitis in dairy cows. Vet Microbiol 136: 142–149. 19058930 10.1016/j.vetmic.2008.10.024

[6] Ruegg PL (2011) Treatment of Clinical Mastitis. Available: http://milkquality.wisc.edu/wp-content/uploads/2011/09/treatment_of_clinical_mastitis.pdf]. Accessed 2013 March.

[7] Bradley AJ, Leach KA, Breen JE, Green LE, Green MJ (2007) Survey of the incidence and aetiology of mastitis on dairy farms in England and Wales. Vet Rec 160: 253–257. 17322356 10.1136/vr.160.8.253

[8] Bradley A (2002) Bovine mastitis: an evolving disease. Vet J 164: 116–128. 12359466 10.1053/tvjl.2002.0724

[9] Fessler AT, Billerbeck C, Kadlec K, Schwarz S (2010) Identification and characterization of methicillin-resistant coagulase-negative staphylococci from bovine mastitis. J Antimicrob Chemother 65: 1576–1582. 20525989 10.1093/jac/dkq172

[10] Krause DO, Hendrick S (2011) Zoonotic Pathogens in the Food Chain. CABI, Oxfordshire, UK. Available: http://www.ssu.ac.ir/fileadmin/templates/fa/daneshkadaha/daneshkade-behdasht/begh/ebook/Zoonotic_Pathogens_in_the_Food_Chain.pdf. Accessed 2012 July.

[11] Gaze W, O’Neill C, Wellington E, Hawkey P (2008) Antibiotic resistance in the environment, with particular reference to MRSA. Adv Appl Microbiol 63: 249–280. 18395130 10.1016/S0065-2164(07)00007-X

[12] Thomson K, Rantala M, Hautala M, Pyorala S, Kaartinen L (2008) Cross-sectional prospective survey to study indication-based usage of antimicrobials in animals: results of use in cattle. BMC Vet Res 4: 15. 18410674 10.1186/1746-6148-4-15 PMC2375862

[13] Pyorala S, Taponen S (2009) Coagulase-negative staphylococci-emerging mastitis pathogens. Vet Microbiol 134: 3–8. 18848410 10.1016/j.vetmic.2008.09.015

[14] Taponen S, Pyorala S (2009) Coagulase-negative staphylococci as cause of bovine mastitis- not so different from Staphylococcus aureus? Vet Microbiol 134: 29–36. 18977615 10.1016/j.vetmic.2008.09.011

[15] Hendriksen RS, Mevius DJ, Schroeter A, Teale C, Meunier D, et al (2008) Prevalence of antimicrobial resistance among bacterial pathogens isolated from cattle in different European countries: 2002–2004. Acta Vet Scand 50: 28. 18611246 10.1186/1751-0147-50-28 PMC2486267

[16] Claesson C (2012) Staphylococci and Enterococci: Studies on activity of antimicrobial agents and detection of genes involved in biofilm formation. Linkoping University. Available: liu.diva-portal.org/smash/get/diva2:352084/FULLTEXT01. Accessed 2012 December.

[17] Carroll KC, Borek AP, Burger C, Glanz B, Bhally H, et al (2006) Evaluation of the BD Phoenix automated microbiology system for identification and antimicrobial susceptibility testing of staphylococci and enterococci. J Clin Microbiol 44: 2072–2077. 16757600 10.1128/JCM.02636-05 PMC1489426

[18] Carroll KC, Glanz BD, Borek AP, Burger C, Bhally HS, et al (2006) Evaluation of the BD Phoenix automated microbiology system for identification and antimicrobial susceptibility testing of Enterobacteriaceae. J Clin Microbiol 44: 3506–3509. 17021074 10.1128/JCM.00994-06 PMC1594749

[19] Laboratory Procedures: BD Phoenix™ PMIC/ID Panels BPPP, BD Phoenix™ PID Panels. Available: http://www.bd.com/. Accessed 2013 January.

[20] O’Hara CM (2006) Evaluation of the Phoenix 100 ID/AST system and NID panel for identification of Enterobacteriaceae, Vibrionaceae, and commonly isolated nonenteric gram-negative bacilli. J Clin Microbiol 44: 928–933. 16517878 10.1128/JCM.44.3.928-933.2006 PMC1393076

[21] Persson Y, Nyman AK, Gronlund-Andersson U (2011) Etiology and antimicrobial susceptibility of udder pathogens from cases of subclinical mastitis in dairy cows in Sweden. Acta Vet Scand 53: 36. 21649936 10.1186/1751-0147-53-36 PMC3118135

[22] Kateete DP, Namazzi S, Okee M, Okeng A, Baluku H, et al (2011) High prevalence of methicillin resistant Staphylococcus aureus in the surgical units of Mulago hospital in Kampala, Uganda. BMC Res Notes 4: 326. 21899769 10.1186/1756-0500-4-326 PMC3184088

[23] Zadoks RN, Middleton JR, McDougall S, Katholm J, Schukken YH (2011) Molecular epidemiology of mastitis pathogens of dairy cattle and comparative relevance to humans. J Mammary Gland Biol Neoplasia 16: 357–372. 21968538 10.1007/s10911-011-9236-y PMC3208832

[24] Cheng DR, Zhu SY, Yin ZH, Ding WW, Mu ZX, et al. (2010) Prevalence of bacterial infection responsible for bovine mastitis. African Journal of Microbiology Research 4: 1110–1116. Available: http://www.academicjournals.org/ajmr/PDF/Pdf2010/4Jun/DaRong%20et%20al.pdf. Accessed 2012 October.

[25] Sumathi BR, Veeregowda BM, Gomes AR (2008) Prevalence and antibiogram profile of bacterial Isolates from clinical bovine mastitis. Veterinary World 1: 237–238. Available: http://veterinaryworld.org/2008/August/Prevalence%20and%20antibiogram%20profile%20of%20bacterial%20httpIsolates.pdf. Accessed 2012 May.

[26] Huijsdens XW, van Dijke BJ, Spalburg E, van Santen-Verheuvel MG, Heck ME, et al (2006) Community-acquired MRSA and pig-farming. Ann Clin Microbiol Antimicrob 5: 26. 17096847 10.1186/1476-0711-5-26 PMC1654169

[27] Shearer JE, Wireman J, Hostetler J, Forberger H, Borman J, et al (2011) Major families of multiresistant plasmids from geographically and epidemiologically diverse staphylococci. G3 (Bethesda) 1: 581–591. 22384369 10.1534/g3.111.000760 PMC3276174

[28] Mayer LW (1988) Use of plasmid profiles in epidemiologic surveillance of disease outbreaks and in tracing the transmission of antibiotic resistance. Clin Microbiol Rev 1: 228–243. 2852997 10.1128/cmr.1.2.228 PMC358044

[29] McCarthy AJ, Lindsay JA (2012) The distribution of plasmids that carry virulence and resistance genes in Staphylococcus aureus is lineage associated. BMC Microbiol 12: 104. 22691167 10.1186/1471-2180-12-104 PMC3406946

[30] Blanco R, Tristan A, Ezpeleta G, Larsen AR, Bes M, et al (2011) Molecular epidemiology of Panton-Valentine leukocidin-positive Staphylococcus aureus in Spain: emergence of the USA300 clone in an autochthonous population. J Clin Microbiol 49: 433–436. 21068288 10.1128/JCM.02201-10 PMC3020469

[31] Seni J (2012) Molecular epidemiology of methicillin resistant Staphylococcus aureus in patients with surgical site infections at Mulago hospital in Kampala, Uganda. MSc Thesis. Makerere University, Kampala.

[32] Bakir M, Sabrina R, Toufik M (2011) Antibacterial susceptibility profiles of sub-clinical mastitis pathogens isolated from cows in Batna and Setif Governorates (East of Algeria). Veterinary World 4: 537–541. Available: http://www.scopemed.org/fulltextpdf.php?mno=12143]. Accessed 2012 November.

[33] Honkanen-Buzalski T, Myllys V, Pyörälä S (1994) Bovine Clinical Mastitis due to Coagulase-negative Staphylococci and their Susceptibility to Antimicrobials. Journal of Veterinary Medicine, Series B 41: 344–350. 7839757 10.1111/j.1439-0450.1994.tb00237.x

[34] Madut NA, Gadir AEA, El Jalii IM (2009) Host determinants of bovine mastitis in semi-intensive production system of Khartoum state, Sudan. Journal of Cell and Animal Biology 3: 71–77. Available: ttp://www.academicjournals.org/jcab/PDF/Pdf2009/May/Madut%20et%20al.pdf. Accessed 2013 February.

[35] Hijazin M, Ulbegi-Mohyla H, Alber J, Lammler C, Hassan AA, et al (2011) Molecular identification and further characterization of Arcanobacterium pyogenes isolated from bovine mastitis and from various other origins. J Dairy Sci 94: 1813–1819. 21426970 10.3168/jds.2010-3678

[36] Kalmus P, Aasmae B, Karssin A, Orro T, Kask K (2011) Udder pathogens and their resistance to antimicrobial agents in dairy cows in Estonia. Acta Vet Scand 53: 4. 21299911 10.1186/1751-0147-53-4 PMC3041692

[37] Huber H, Ziegler D, Pfluger V, Vogel G, Zweifel C, et al (2011) Prevalence and characteristics of methicillin-resistant coagulase-negative staphylococci from livestock, chicken carcasses, bulk tank milk, minced meat, and contact persons. BMC Vet Res 7: 6. 21272304 10.1186/1746-6148-7-6 PMC3042402

[38] Uganda National Council of Science and Technology (UNCST) (2007) National guidelines for research involving humans as research participants. Available: http://www.uncst.go.ug/index.php/about-us.html. Accessed 2012 September.

[39] Makita K, Fevre EM, Waiswa C, Eisler MC, Thrusfield M, et al (2011) Herd prevalence of bovine brucellosis and analysis of risk factors in cattle in urban and peri-urban areas of the Kampala economic zone, Uganda. BMC Vet Res 7: 60. 22004574 10.1186/1746-6148-7-60 PMC3212899

[40] Kateete DP, Kimani CN, Katabazi FA, Okeng A, Okee MS, et al (2010) Identification of Staphylococcus aureus: DNase and Mannitol salt agar improve the efficiency of the tube coagulase test. Ann Clin Microbiol Antimicrob 9: 23. 20707914 10.1186/1476-0711-9-23 PMC2927478

[41] Devriese LA, Van De Kerckhove A, Kilpper-BÃ¤lz R, Schleifer KH (1987) Characterization and Identification of Enterococcus Species Isolated from the Intestines of Animals. International Journal of Systematic Bacteriology 37: 257–259.

[42] The Enterobacteriaceae - In color Atlas and Textbook of diagnostic Microbiology: Koneman EW AS, Janda WM, Schreckenberger PC, Winn Jr. WC (editors). Philadelphia: JB Lippincott Company; 5th edition, 1997. p. 171–252.

[43] Harmsen D, Claus H, Witte W, Rothganger J, Turnwald D, et al (2003) Typing of methicillin-resistant Staphylococcus aureus in a university hospital setting by using novel software for spa repeat determination and database management. J Clin Microbiol 41: 5442–5448. 14662923 10.1128/JCM.41.12.5442-5448.2003 PMC309029

[44] Reinoso E, Bettera S, Frigerio C, DiRenzo M, Calzolari A, et al (2004) RAPD-PCR analysis of Staphylococcus aureus strains isolated from bovine and human hosts. Microbiol Res 159: 245–255. 15462524 10.1016/j.micres.2004.04.002

