# Supplementary material for: Prevalence and Antimicrobial Susceptibility Patterns of Bacteria from Milkmen and Cows with Clinical Mastitis in and around Kampala, Uganda
Source: PLoS One. 2013 May 7;8(5):e63413. doi: 10.1371/journal.pone.0063413 (PMC3646745; doi:10.1371/journal.pone.0063413)
Supplement: Table S2 — Antimicrobial resistance patterns of each staphylococcal isolate. (PDF) [file pone.0063413.s002.pdf]

Table S2: Antimicrobial resistance patterns among Staphylococci

Isolates from bovine samples (n = 21)		
**Species**	**Antimicrobial resistance pattern**	**Comment***
S. hyicus	AMP-PEN-SXT-TET	
S. hyicus	AMP-PEN-CEF-OXA-AMO	MRS
S. hyicus	AMP-PEN-CEF-OXA-AMO	MRS
S. hyicus	AMP-PEN-CEF-OXA-AMO	MRS
S. saprophyticus	AMP-PEN-SXT	
S. saprophyticus	AMP-PEN-SXT-TET-CEF-OXA-AMO-ERY	MRS, MLS_B
S. saprophyticus	AMP-PEN-CEF-OXA-AMO	MRS
S. saprophyticus	AMP-PEN-CEF-OXA-AMO	MRS
S. xylosus	AMP-PEN-SXT-TET-CEF-OXA-CLI-ERY	MRS, MLS_B, STAIML
S. xylosus	AMP-PEN-SXT	
S. xylosus	AMP-PEN-TET	
S. *scuiri*	AMP-PEN-CEF-OXA-CLI	MRS, STAIML
S. *scuiri*	AMP-PEN	
S. aureus	AMP-PEN	
S. epidermis	AMP-PEN-SXT-TET	
S. haemolyticus	AMP-PEN	
S. hominis	AMP-PEN-CEF-OXA-AMO-TEI-VAN-NTR-RIF	MR-VRS
S. lugdunensis	AMP-PEN-TET-CEF-OXA-AMO-TEI-VAN-NTR-RIF	MR-VRS
S. gallinarum	AMP-PEN-TET-CEF-OXA-AMO-CLI	MRS, STAIML
S. pasteuri	AMP-PEN-TET	
S. intermedius	AMP-PEN-CEF-OXA-AMO	MRS
**Isolates from human nares (n = 11)**		
S. aureus	AMP-PEN-SXT-TET	
S. aureus	AMP-PEN-SXT-TET	
S. aureus	AMP-PEN-SXT-TET	
S. aureus	AMP-PEN-SXT-TET	
**S. *scuiri***	AMP-PEN-CEF-OXA-AMO-TEI-VAN-CLI-ERY-NTR-CIP	STAIML, MLS_B, MR-VRS
**S. *scuiri***	AMP-PEN-CEF-OXA-AMO-TEI-VAN-CLI-ERY-NTR-CIP	STAIML, MLS_B, MR-VRS
**S. *scuiri***	AMP-PEN-CEF-OXA-AMO-TEI-VAN-CLI-ERY-NTR-CIP	STAIML, MLS_B, MR-VRS
S. saprophyticus	AMP-PEN-SXT-TET-CEF-OXA-AMO	MRS
S. saprophyticus	AMP-PEN-CEF-OXA-AMO	MRS
S. xylosus	AMP-PEN-SXT-TET-CEF-OXA-AMO-CLI-ERY	MRS, STAIML, MLS_B
S. intermedius	AMP-PEN-SXT-TET-CEF-OXA-AMO-CLI-ERY	MRS, STAIML, MLS_B

AMP, Ampicillin; PEN, Penicillin G; SXT, trimethopprim-sulfamethoxazole; TET, tetracycline; CEF, Cefoxitine; OXA, Oxacillin; AMO; Amoxicillin-Clavulanate; TEI , Teicoplanin; VAN, Vancomycin; CLI, Clindamycin; ERY, Erythromycin; NTR, Nitrofurantoin; RIF, Rifampicin; CIP, Ciprofloxacin

MRS, Methicillin resistant staphylococcus; STAIML, Staphylococcus inducible MLS_B phenotype; MLS_B, resistance to Macrolides, Lincosamide and Streptogramins

In boldface type are isolates found to be concomitantly methicillin and vancomycin resistant (i.e., MR-VRS, methicillin resistant-vancomycin resistant staphylococci)

*****All staphylococci were Beta-lactamase producers
