# Supplementary material for: Prevalence and Antimicrobial Susceptibility Patterns of Bacteria from Milkmen and Cows with Clinical Mastitis in and around Kampala, Uganda
Source: PLoS One. 2013 May 7;8(5):e63413. doi: 10.1371/journal.pone.0063413 (PMC3646745; doi:10.1371/journal.pone.0063413)
Supplement: Table S3 — Primers for genotyping daptomycin and vancomycin resistant enterococci. (PDF) [file pone.0063413.s003.pdf]

Table S2: Primers for MLST of *Enterococcus faecium*

The following primers were used to amplify the seven house-keeping gene fragments (1 is the forward and 2 the reversed primer).

adk1n:	5’-GAACCTCATTTTAATGGGG-3’
adk2n:	5’-TGATGTTGATAGCCAGACG-3’
atpA1n:	5’-TTCAAATGGCTCATACGG-3’
atpA2n:	5’-AGTTCACGATAAGCAACAGC-3’
ddl1:	5’-GAGACATTGAATATGCCTTATG-3’
ddl2:	5’-AAAAAGAAATCGCACCG-3’
gdh1:	5’-GGCGCACTAAAAGATATGGT-3’
gdh2:	5’-CCAAGATTGGGCAACTTCGTCCCA-3’
gyd-1:	5’-CAAACTGCTTAGCTCCAATGGC-3’
gyd2:	5’-CATTTCGTTGTCATACCAAGC-3’
purK1n:	5’-CAGATTGGCACATTGAAAG-3’
purK2n:	5’-TTCATTCACATATAGCCCG-3’
pstS1n:	5’-TTGAGCCAAGTCGAAGC-3’
pstS2:	5’-CGTGATCACGTTCTACTTCC-3’

From: http://efaecium.mlst.net/misc/info.asp

Primers for MLST of *Enterococcus faecalis*

Genes and Function	Sequences (5'-3')	Size of amplicon used for assigning alleles
**glucose-6-phosphate dehydrogenase**		
gdh-1	GGCGCACTAAAAGATATGGT	530
gdh-2	CCAAGATTGGGCAACTTCGTCCCA	
**glyceraldehydes-3-phosphate dehydrogenase**		
gyd-1	CAAACTGCTTAGCTCCAATGGC	395
gyd-2	CATTTCGTTGTCATACCAAGC	
**phosphate ATP binding cassette transporter**		
pstS-1	CGGAACAGGACTTTCGC	583
pstS-2	ATTTACATCACGTTCTACTTGC	
**Glucokinase**		
gki-1	GATTTTGTGGGAATTGGTATGG	438
gki-2	ACCATTAAAGCAAAATGATCGC	
**shikimate-5-dehydrogenase**		
aroE-1	TGGAAAACTTTACGGAGACAGC	459
aroE-2	GTCCTGTCCATTGTTCAAAAGC	
**xanthine phosphoribosyltransferase**		
xpt-1	AAAATGATGGCCGTGTATTAGG	456
xpt-2	AACGTCACCGTTCCTTCACTTA	
**acetyl-CoA acetyltransferase**		
yiqL-1	CAGCTTAAGTCAAGTAAGTGCCG	436
yiqL-2	GAATATCCCTTCTGCTTGTGCT	

From: http://efaecalis.mlst.net/misc/info.asp
